# Supplementary material for: Autistic traits influence the strategic diversity of information sampling: Insights from two-stage decision models
Source: PLoS Comput Biol. 2019 Dec 2;15(12):e1006964. doi: 10.1371/journal.pcbi.1006964 (PMC6907874; doi:10.1371/journal.pcbi.1006964)
Supplement: S1 Table — (PDF) [file pcbi.1006964.s001.pdf]

S1 Table. Model descriptions.

Model	One- or two-stage?	Cost-first or evidence-first?	Continue-then-2nd-thought or stop-then-2nd-thought?	Second-thought probabilities	Number of parameters for sampling choice	Number of parameters for decision time	Cost-related decision variables	Evidence-related decision variables	Decay parameter
Cost only	One-stage	N/A	N/A	N/A	5	4	A constant, unit cost (categorical, 3 levels), the number of beads sampled, total sampling cost	NA	NA
Evidence only w/o decay	One-stage	N/A	N/A	N/A	6	4	NA	A constant, unit log evidence, absolute value of cumulative information, total log evidence	NA
Cost + Evidence w/o decay	One-stage	N/A	N/A	N/A	10	4	As in *Cost only* model	As in Evidence only w/o decay	NA
Cost + Evidence	One-stage	N/A	N/A	N/A	11	4	As in *Cost only* model	A constant, unit log evidence, absolute value of decayed cumulative information, total decayed log evidence	α
Cost $\xrightarrow[\text{C-cond}]{\text{continue}}$ Evidence	Two-stage	Cost-related variables in the first step	Continue sampling in the first step then having the second thought	Controlled by cost conditions	15	8	As in *Cost only* model	As in *Cost + Evidence* model	α

Cost $\xrightarrow[\text{E-cond}]{\text{continue}}$ Evidence	Two-stage	Cost-related variables in the first step	Continue sampling in the first step then having the second thought	Controlled by evidence conditions	14	8	As in *Cost only* model	As in *Cost + Evidence* model	α
Cost $\xrightarrow[\text{flex}]{\text{continue}}$ Evidence	Two-stage	Cost-related variables in the first step	Continue sampling in the first step then having the second thought	Controlled by the probability of stop in the first stage	14	8	As in *Cost only* model	As in *Cost + Evidence* model	α
Cost $\xrightarrow[\text{C-cond}]{\text{stop}}$ Evidence	Two-stage	Cost-related variables in the first step	Stop sampling in the first step then having the second thought	Controlled by cost conditions	15	8	As in *Cost only* model	As in *Cost + Evidence* model	α
Cost $\xrightarrow[\text{E-cond}]{\text{stop}}$ Evidence	Two-stage	Cost-related variables in the first step	Stop sampling in the first step then having the second thought	Controlled by evidence conditions	14	8	As in *Cost only* model	As in *Cost + Evidence* model	α
Cost $\xrightarrow[\text{flex}]{\text{stop}}$ Evidence	Two-stage	Cost-related variables in the first step	Stop sampling in the first step then having the second thought	Controlled by the probability of stop in the first stage	14	8	As in *Cost only* model	As in *Cost + Evidence* model	α
Evidence $\xrightarrow[\text{C-cond}]{\text{continue}}$ Cost	Two-stage	Evidence-related variables in the first step	Continue sampling in the first step then having the second thought	Controlled by cost conditions	15	8	As in *Cost only* model	As in *Cost + Evidence* model	α

Evidence $\xrightarrow[\text{E-cond}]{\text{continue}}$ Cost	Two-stage	Evidence-related variables in the first step	Continue sampling in the first step then having the second thought	Controlled by evidence conditions	14	8	As in *Cost only* model	As in *Cost* + *Evidence* model	α
Evidence $\xrightarrow[\text{flex}]{\text{continue}}$ Cost	Two-stage	Evidence-related variables in the first step	Continue sampling in the first step then having the second thought	Controlled by the probability of stop in the first stage	14	8	As in *Cost only* model	As in *Cost* + *Evidence* model	α
Evidence $\xrightarrow[\text{C-cond}]{\text{stop}}$ Cost	Two-stage	Evidence-related variables in the first step	Stop sampling in the first step then having the second thought	Controlled by cost conditions	15	8	As in *Cost only* model	As in *Cost* + *Evidence* model	α
Evidence $\xrightarrow[\text{E-cond}]{\text{stop}}$ Cost	Two-stage	Evidence-related variables in the first step	Stop sampling in the first step then having the second thought	Controlled by evidence conditions	14	8	As in *Cost only* model	As in *Cost* + *Evidence* model	α
Evidence $\xrightarrow[\text{flex}]{\text{stop}}$ Cost	Two-stage	Evidence-related variables in the first step	Stop sampling in the first step then having the second thought	Controlled by the probability of stop in the first stage	14	8	As in *Cost only* model	As in *Cost* + *Evidence* model	α
