# Supplementary material for: Autistic traits influence the strategic diversity of information sampling: Insights from two-stage decision models
Source: PLoS Comput Biol. 2019 Dec 2;15(12):e1006964. doi: 10.1371/journal.pcbi.1006964 (PMC6907874; doi:10.1371/journal.pcbi.1006964)
Supplement: S1 Fig — Scatter plots of AQ-related effects of interest: efficiency (a), sampling bias (b), and sampling variability (c). Each panel is for one cost and evidence condition. Each semi-transparent datapoint represents one participant. The blue line indicates the regression line against AQ, whose slope corresponds to the ΒAQ in Fig 2D–2F. (PDF) [file pcbi.1006964.s002.pdf]

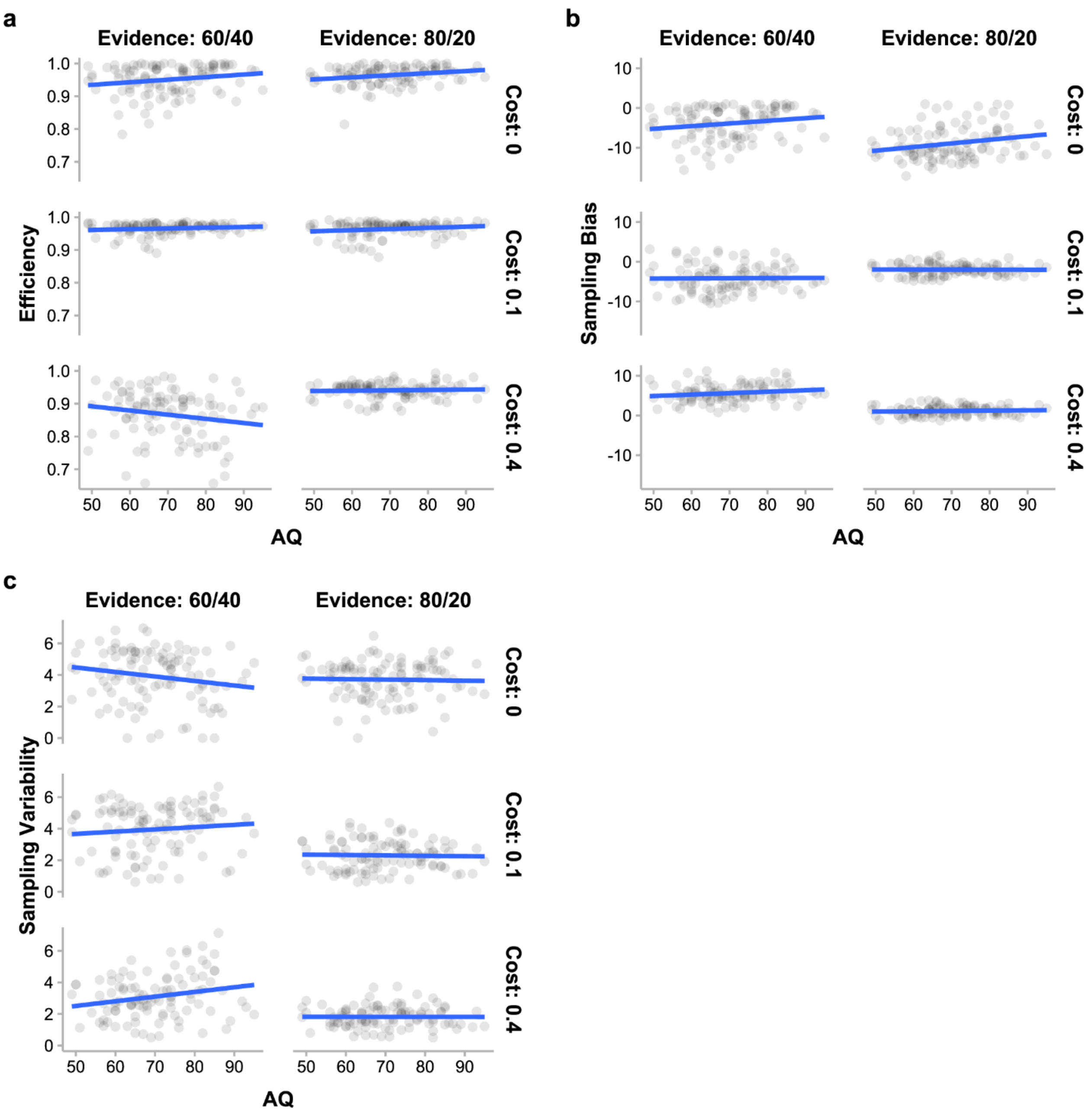

S1 Fig. Scatter plots of AQ-related effects of interest: efficiency (a), sampling bias (b), and sampling variability (c).

Each panel is for one cost and evidence condition. Each semi-transparent datapoint represents one participant. The blue line indicates the regression line against AQ, whose slope corresponds to the B_{AQ} in Fig 2d–f.
