# Supplementary material for: Autistic traits influence the strategic diversity of information sampling: Insights from two-stage decision models
Source: PLoS Comput Biol. 2019 Dec 2;15(12):e1006964. doi: 10.1371/journal.pcbi.1006964 (PMC6907874; doi:10.1371/journal.pcbi.1006964)
Supplement: S2 Fig — (a) Similar to what we have found for the linear effect of AQ on efficiency, we have found a significant three-way interaction of AQ groups, cost and evidence conditions (F4,200.83 = 11.03, p < .001). The simple main effect showed the group with high AQ had higher efficiency in the zero-cost, low-evidence condition, compared to the other two groups with low and middle AQ scores (F2,132.62 = 4.27, p = .016; post hoc comparison: Low–Mid: t132.62 = 0.53, p = .86, Low–High: t132.62 = -2.23, p = .07, Mid–High: t132.62 = -2.77, p = .018, ps were corrected by single-step adjustment, see Methods). Meanwhile, the group with high AQ had significantly lower efficiency in the high-cost, low-evidence condition (simple main effect: F2,117.67 = 8.18, p < .001; post hoc comparison: Low–Mid: t118.45 = -1.60, p = .25, Low–High: t117.29 = 2.42, p = .044, Mid–High: t117.25 = 4.02, p < .001). All these results were consistent with those reported in the main text based on regressions (see Fig 2B). (b) Participants with different levels of autistic traits significantly differed in cost-evidence strategy index (i.e., AICccost→evidence−AICcevidence→cost; F2,101 = 5.96, p = .004), with the value of the high-AQ group smaller than those of the low-AQ group (t101 = -2.81, p = .017) and the middle-AQ group (t101 = -3.175, p = .006). This is consistent with the negative correlation between AQ and cost-evidence strategy index (see Fig 6A). In both (a) and (b), colored lines represent group means and semi-transparent gray symbols represent individual participants. Different shapes of symbols are for different AQ groups: circles for low-AQ, triangles for middle-AQ, and squares for high-AQ. Error bars denote model-based standard errors. Dark orange asterisks and lines indicate significant simple main effects (p < .05). (PDF) [file pcbi.1006964.s003.pdf]

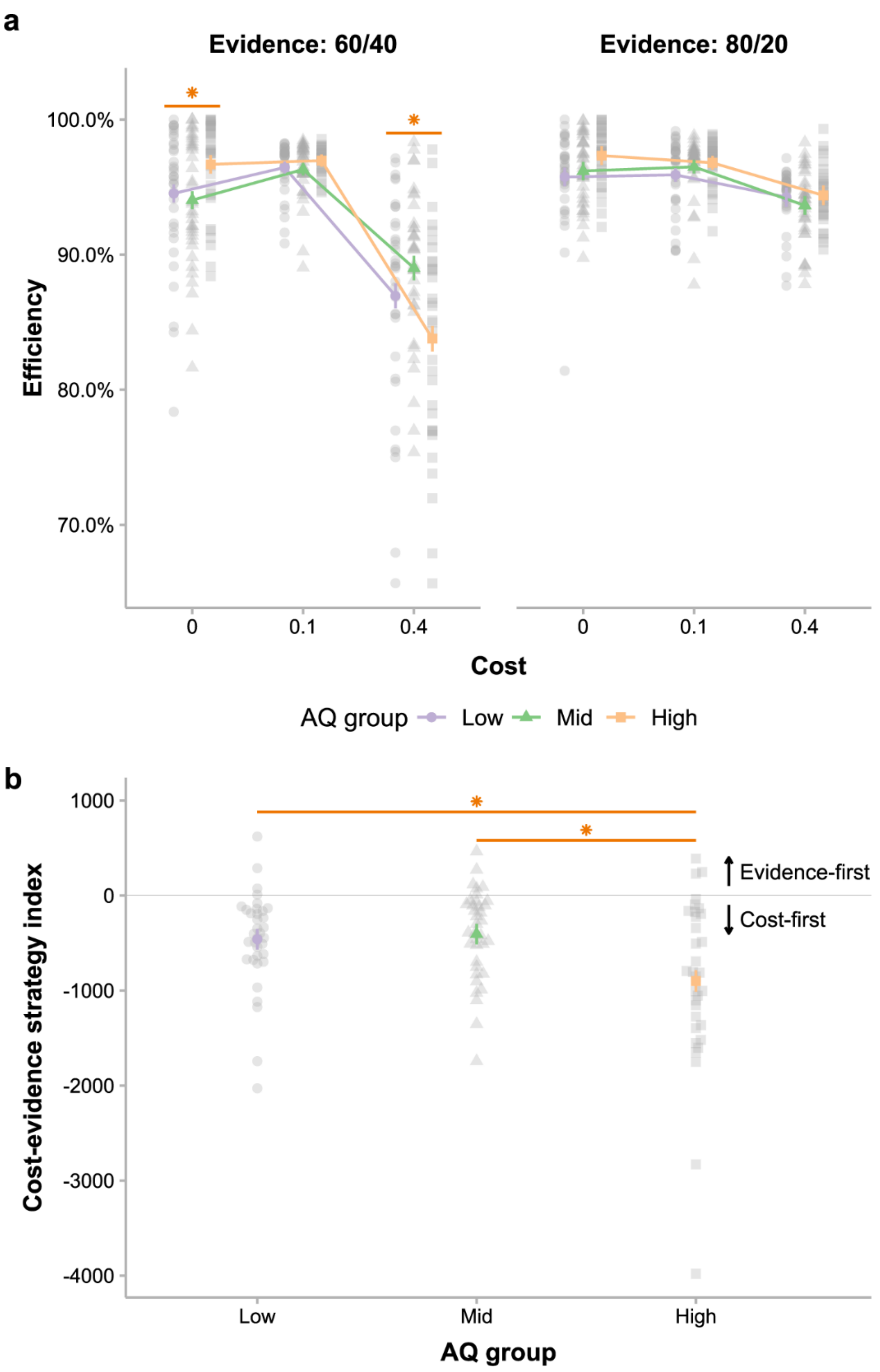

S2 Fig. Replication of two analyses in the main text based on the tripartite division of AQ. (a) Similar to what we have found for the linear effect of AQ on efficiency, we have found a significant three-way interaction of AQ groups, cost and evidence conditions ($F_{4,200.83} = 11.03$, $p < .001$). The simple main effect showed the group with high AQ had higher efficiency in the zero-cost, low-evidence condition, compared to the other two groups with low and middle AQ scores ($F_{2,132.62} = 4.27$, $p = .016$; *post hoc* comparison: Low – Mid: $t_{132.62} = 0.53$, $p = .86$, Low –

High: $t_{132.62} = -2.23$, $p = .07$, Mid – High: $t_{132.62} = -2.77$, $p = .018$, ps were corrected by single-step adjustment, see Methods). Meanwhile, the group with high AQ had significantly lower efficiency in the high-cost, low-evidence condition (simple main effect: $F_{2,117.67} = 8.18$, $p < .001$; *post hoc* comparison: Low – Mid: $t_{118.45} = -1.60$, $p = .25$, Low – High: $t_{117.29} = 2.42$, $p = .044$, Mid – High: $t_{117.25} = 4.02$, $p < .001$). All these results were consistent with those reported in the main text based on regressions (see Fig 2b). (b) Participants with different levels of autistic traits significantly differed in cost-evidence strategy index (i.e., $AICc_{\mathrm{cost} \rightarrow \mathrm{evidence}} - AICc_{\mathrm{evidence} \rightarrow \mathrm{cost}}$; $F_{2,101}=5.96$, $p = .004$), with the value of the high-AQ group smaller than those of the low-AQ group ($t_{101} = -2.81$, $p = .017$) and the middle-AQ group ($t_{101}=-3.175$, $p = .006$). This is consistent with the negative correlation between AQ and cost-evidence strategy index (see Fig 6a). In both (a) and (b), colored lines represent group means and semi-transparent gray symbols represent individual participants. Different shapes of symbols are for different AQ groups: circles for low-AQ, triangles for middle-AQ, and squares for high-AQ. Error bars denote model-based standard errors. Dark orange asterisks and lines indicate significant simple main effects ($p < .05$).
