# Supplementary material for: Autistic traits influence the strategic diversity of information sampling: Insights from two-stage decision models
Source: PLoS Comput Biol. 2019 Dec 2;15(12):e1006964. doi: 10.1371/journal.pcbi.1006964 (PMC6907874; doi:10.1371/journal.pcbi.1006964)
Supplement: S4 Fig — The observed decision times had a significant decreasing trend with the increase of sample number (t = -12.26, p < .001), which was captured by the best two-stage model (red dots) but not by the best one-stage model (blue dots). (PDF) [file pcbi.1006964.s005.pdf]

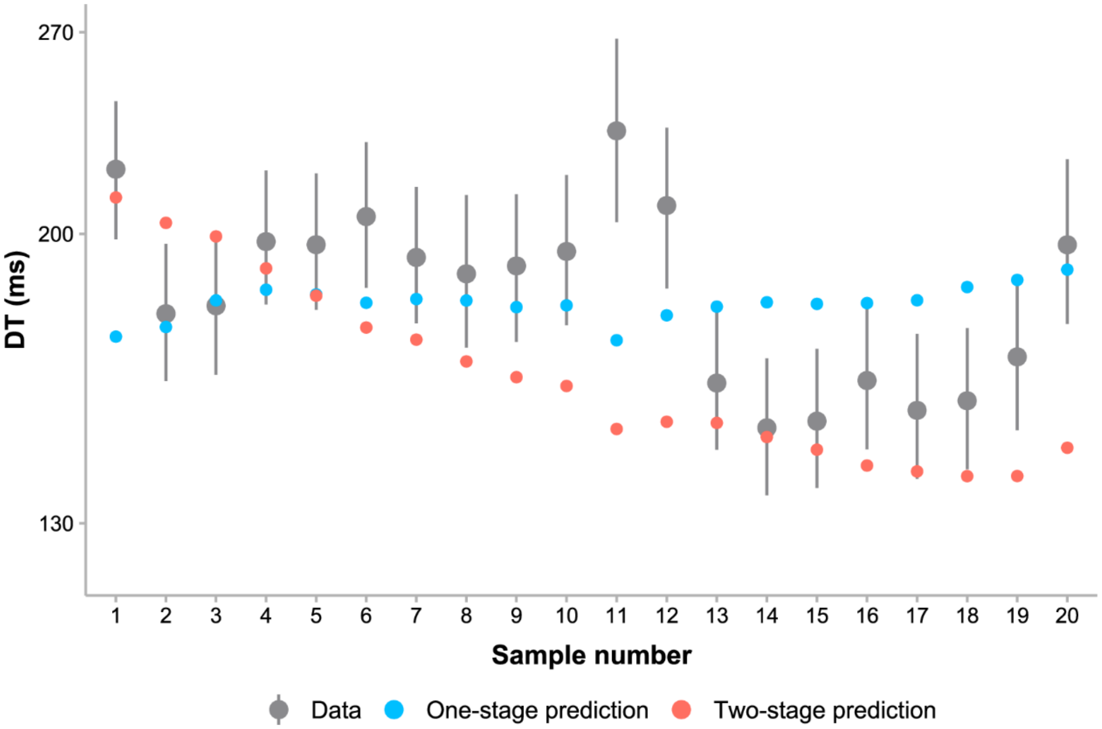

S4 Fig. Decision time as a function of sample number: data vs. model predictions.
The observed decision times had a significant decreasing trend with the increase of sample number (t = -12.26, p < .001), which was captured by the best two-stage model (red dots) but not by the best one-stage model (blue dots).
