# Supplementary material for: Autistic traits influence the strategic diversity of information sampling: Insights from two-stage decision models
Source: PLoS Comput Biol. 2019 Dec 2;15(12):e1006964. doi: 10.1371/journal.pcbi.1006964 (PMC6907874; doi:10.1371/journal.pcbi.1006964)
Supplement: S5 Fig — (a-b) Mean ΔAICc and ΔBIC for every model. ΔAICc or ΔBIC for a specific model was calculated for each participant with respect to the participant’s best-fitting model (i.e. lowest AICc or BIC) and then averaged across participants. Error bars denote standard errors. Model comparisons based on AICc and BIC led to almost the same results. (c-d) Individual participants’ ΔAICc and ΔBIC for every model. In the heatmaps, each column is for one participant, arranged in ascending order of AQ from left to right. Each row is for one model, arranged in the same order as in a-b. (PDF) [file pcbi.1006964.s006.pdf]

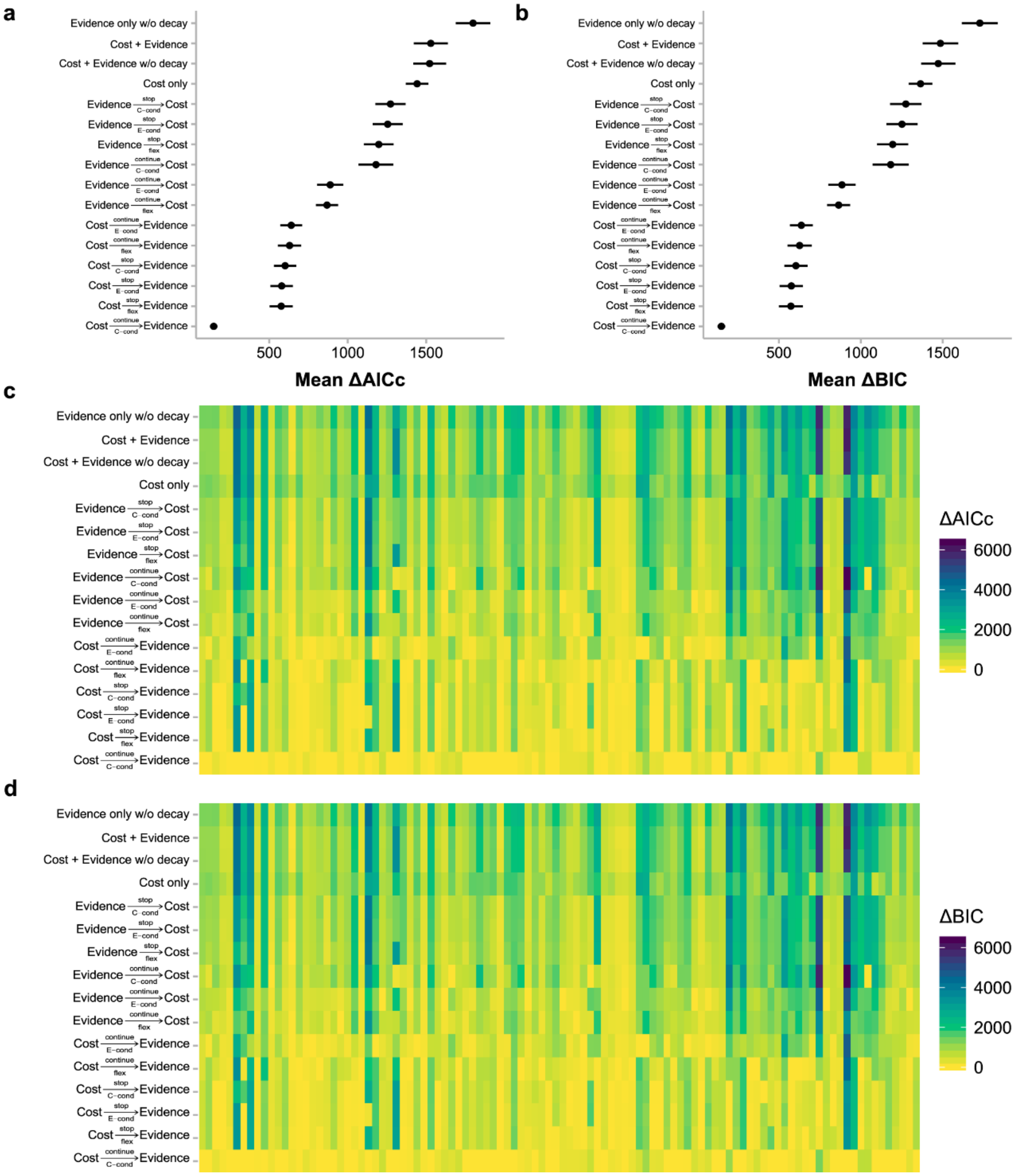

S5 Fig. Model comparisons based on AICc and BIC and individual participants' model evidence.

(a-b) Mean ΔAICc and ΔBIC for every model. ΔAICc or ΔBIC for a specific model was calculated for each participant with respect to the participant's best-fitting model (i.e. lowest AICc or BIC) and then averaged across participants. Error bars denote standard errors. Model comparisons based on AICc and BIC led to almost the same results. (c-d) Individual participants' ΔAICc and ΔBIC for every model. In the heatmaps, each column is for one participant, arranged in ascending order of AQ from left to right. Each row is for one model, arranged in the same order as in a-b.
