# Supplementary material for: Autistic traits influence the strategic diversity of information sampling: Insights from two-stage decision models
Source: PLoS Comput Biol. 2019 Dec 2;15(12):e1006964. doi: 10.1371/journal.pcbi.1006964 (PMC6907874; doi:10.1371/journal.pcbi.1006964)
Supplement: S6 Fig — Gray, blue, and red lines respectively denote data, the best one-stage model predictions, and the best two-stage model predictions. Each panel is for one participant, with each of its sub-panels for one cost and evidence condition. Panels are arranged by participants’ AQ (marked at the top-left corner) ascendingly from left to right and from top to bottom. For most participants, the observed sample size distributions were better predicted by the best-fit one-stage model than by the best-fit one-stage model. C: Cost, E: Evidence. (PDF) [file pcbi.1006964.s007.pdf]

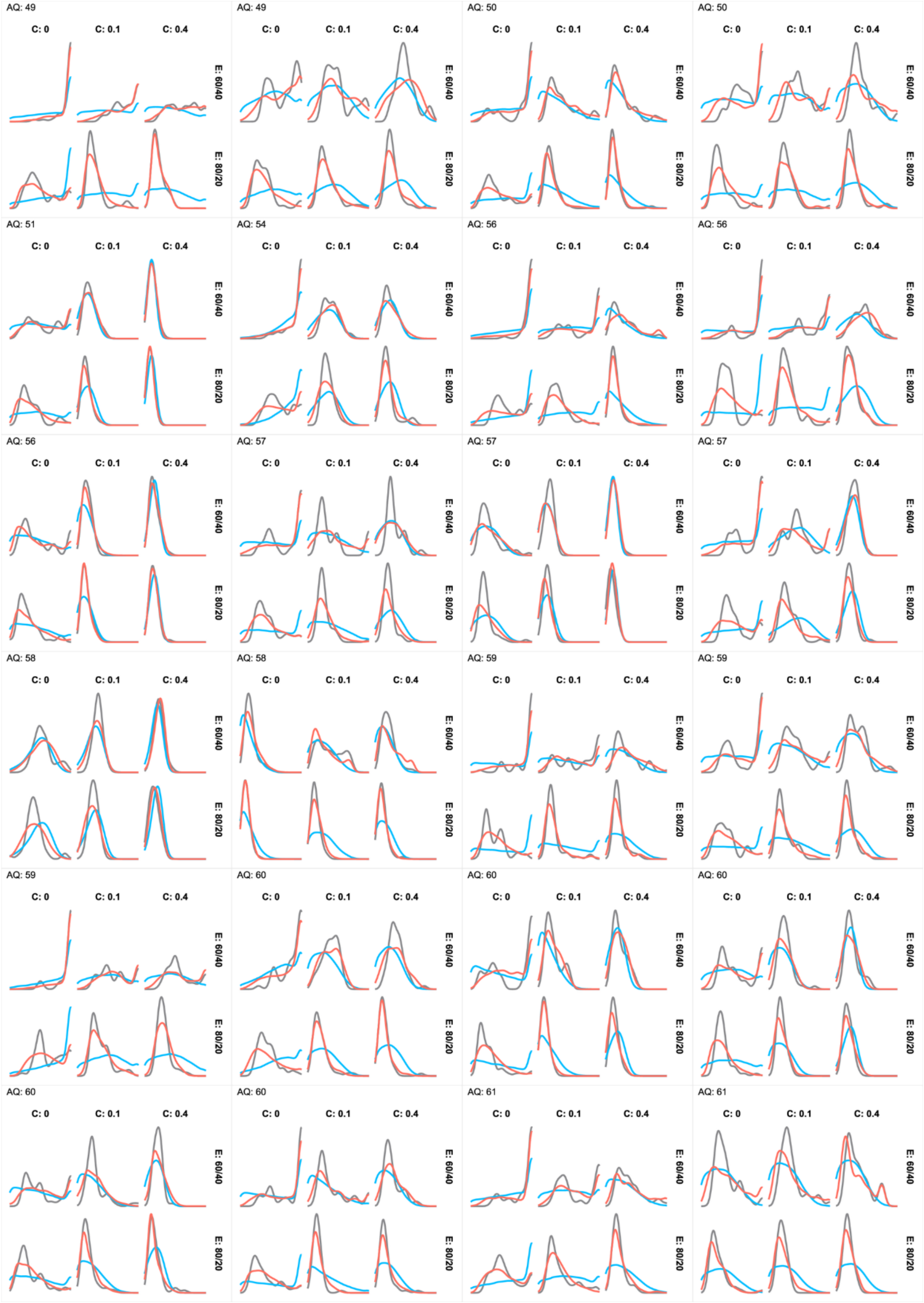
AQ: 49
C: 0
C: 0.1
C: 0.4
E: 60/40
E: 80/20
AQ: 49
C: 0
C: 0.1
C: 0.4
E: 60/40
E: 80/20
AQ: 50
C: 0
C: 0.1
C: 0.4
E: 60/40
E: 80/20
AQ: 50
C: 0
C: 0.1
C: 0.4
E: 60/40
E: 80/20
AQ: 51
C: 0
C: 0.1
C: 0.4
E: 60/40
E: 80/20
AQ: 54
C: 0
C: 0.1
C: 0.4
E: 60/40
E: 80/20
AQ: 56
C: 0
C: 0.1
C: 0.4
E: 60/40
E: 80/20
AQ: 56
C: 0
C: 0.1
C: 0.4
E: 60/40
E: 80/20
AQ: 56
C: 0
C: 0.1
C: 0.4
E: 60/40
E: 80/20
AQ: 57
C: 0
C: 0.1
C: 0.4
E: 60/40
E: 80/20
AQ: 57
C: 0
C: 0.1
C: 0.4
E: 60/40
E: 80/20
AQ: 57
C: 0
C: 0.1
C: 0.4
E: 60/40
E: 80/20
AQ: 58
C: 0
C: 0.1
C: 0.4
E: 60/40
E: 80/20
AQ: 58
C: 0
C: 0.1
C: 0.4
E: 60/40
E: 80/20
AQ: 59
C: 0
C: 0.1
C: 0.4
E: 60/40
E: 80/20
AQ: 59
C: 0
C: 0.1
C: 0.4
E: 60/40
E: 80/20
AQ: 59
C: 0
C: 0.1
C: 0.4
E: 60/40
E: 80/20
AQ: 60
C: 0
C: 0.1
C: 0.4
E: 60/40
E: 80/20
AQ: 60
C: 0
C: 0.1
C: 0.4
E: 60/40
E: 80/20
AQ: 60
C: 0
C: 0.1
C: 0.4
E: 60/40
E: 80/20
AQ: 60
C: 0
C: 0.1
C: 0.4
E: 60/40
E: 80/20
AQ: 60
C: 0
C: 0.1
C: 0.4
E: 60/40
E: 80/20
AQ: 61
C: 0
C: 0.1
C: 0.4
E: 60/40
E: 80/20
AQ: 61
C: 0
C: 0.1
C: 0.4
E: 60/40
E: 80/20

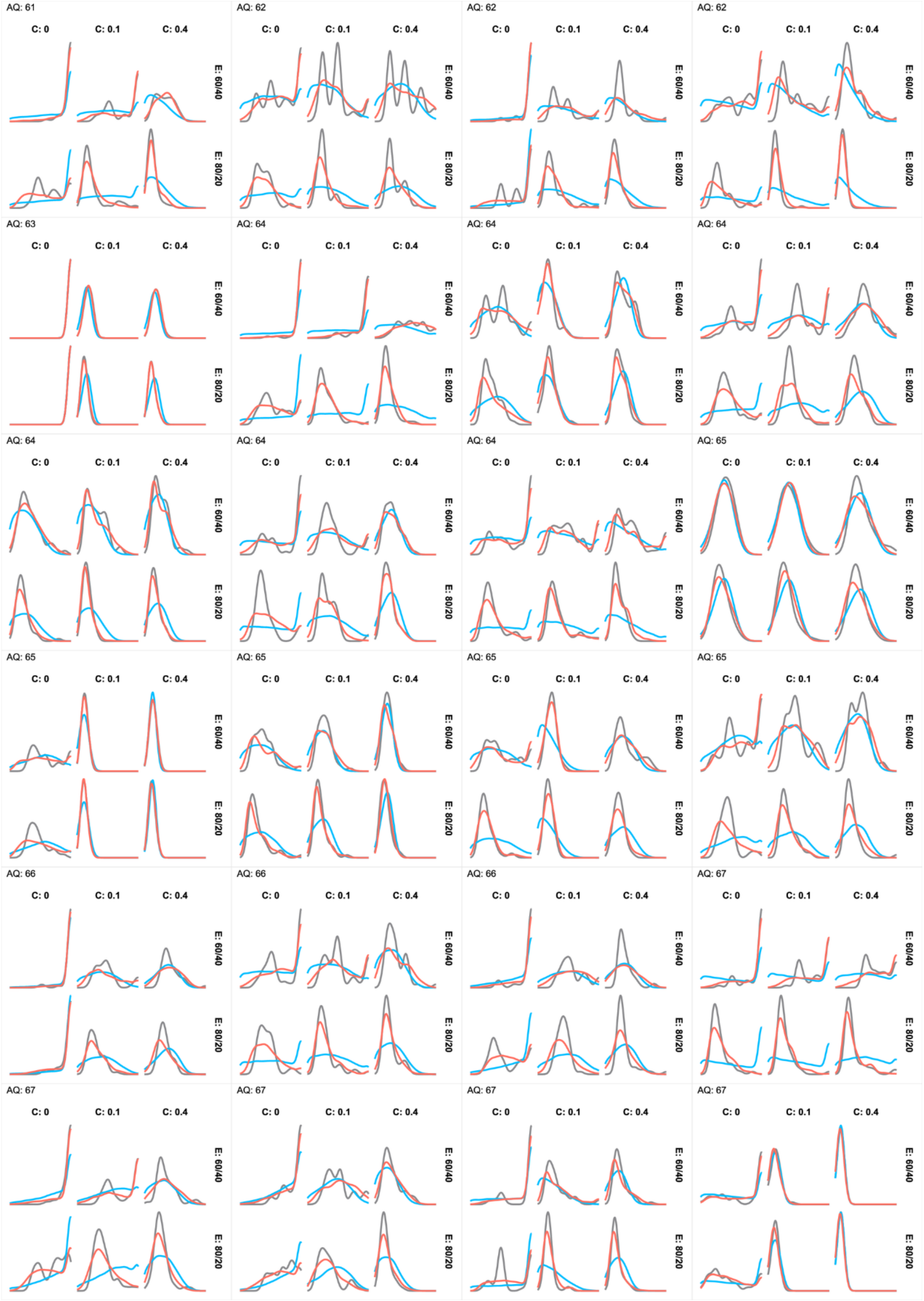
AQ: 61
C: 0
C: 0.1
C: 0.4
E: 60/40
E: 80/20
AQ: 62
C: 0
C: 0.1
C: 0.4
E: 60/40
E: 80/20
AQ: 62
C: 0
C: 0.1
C: 0.4
E: 60/40
E: 80/20
AQ: 62
C: 0
C: 0.1
C: 0.4
E: 60/40
E: 80/20
AQ: 63
C: 0
C: 0.1
C: 0.4
E: 60/40
E: 80/20
AQ: 64
C: 0
C: 0.1
C: 0.4
E: 60/40
E: 80/20
AQ: 64
C: 0
C: 0.1
C: 0.4
E: 60/40
E: 80/20
AQ: 64
C: 0
C: 0.1
C: 0.4
E: 60/40
E: 80/20
AQ: 64
C: 0
C: 0.1
C: 0.4
E: 60/40
E: 80/20
AQ: 64
C: 0
C: 0.1
C: 0.4
E: 60/40
E: 80/20
AQ: 64
C: 0
C: 0.1
C: 0.4
E: 60/40
E: 80/20
AQ: 65
C: 0
C: 0.1
C: 0.4
E: 60/40
E: 80/20
AQ: 65
C: 0
C: 0.1
C: 0.4
E: 60/40
E: 80/20
AQ: 65
C: 0
C: 0.1
C: 0.4
E: 60/40
E: 80/20
AQ: 65
C: 0
C: 0.1
C: 0.4
E: 60/40
E: 80/20
AQ: 65
C: 0
C: 0.1
C: 0.4
E: 60/40
E: 80/20
AQ: 66
C: 0
C: 0.1
C: 0.4
E: 60/40
E: 80/20
AQ: 66
C: 0
C: 0.1
C: 0.4
E: 60/40
E: 80/20
AQ: 66
C: 0
C: 0.1
C: 0.4
E: 60/40
E: 80/20
AQ: 67
C: 0
C: 0.1
C: 0.4
E: 60/40
E: 80/20
AQ: 67
C: 0
C: 0.1
C: 0.4
E: 60/40
E: 80/20
AQ: 67
C: 0
C: 0.1
C: 0.4
E: 60/40
E: 80/20
AQ: 67
C: 0
C: 0.1
C: 0.4
E: 60/40
E: 80/20
AQ: 67
C: 0
C: 0.1
C: 0.4
E: 60/40
E: 80/20

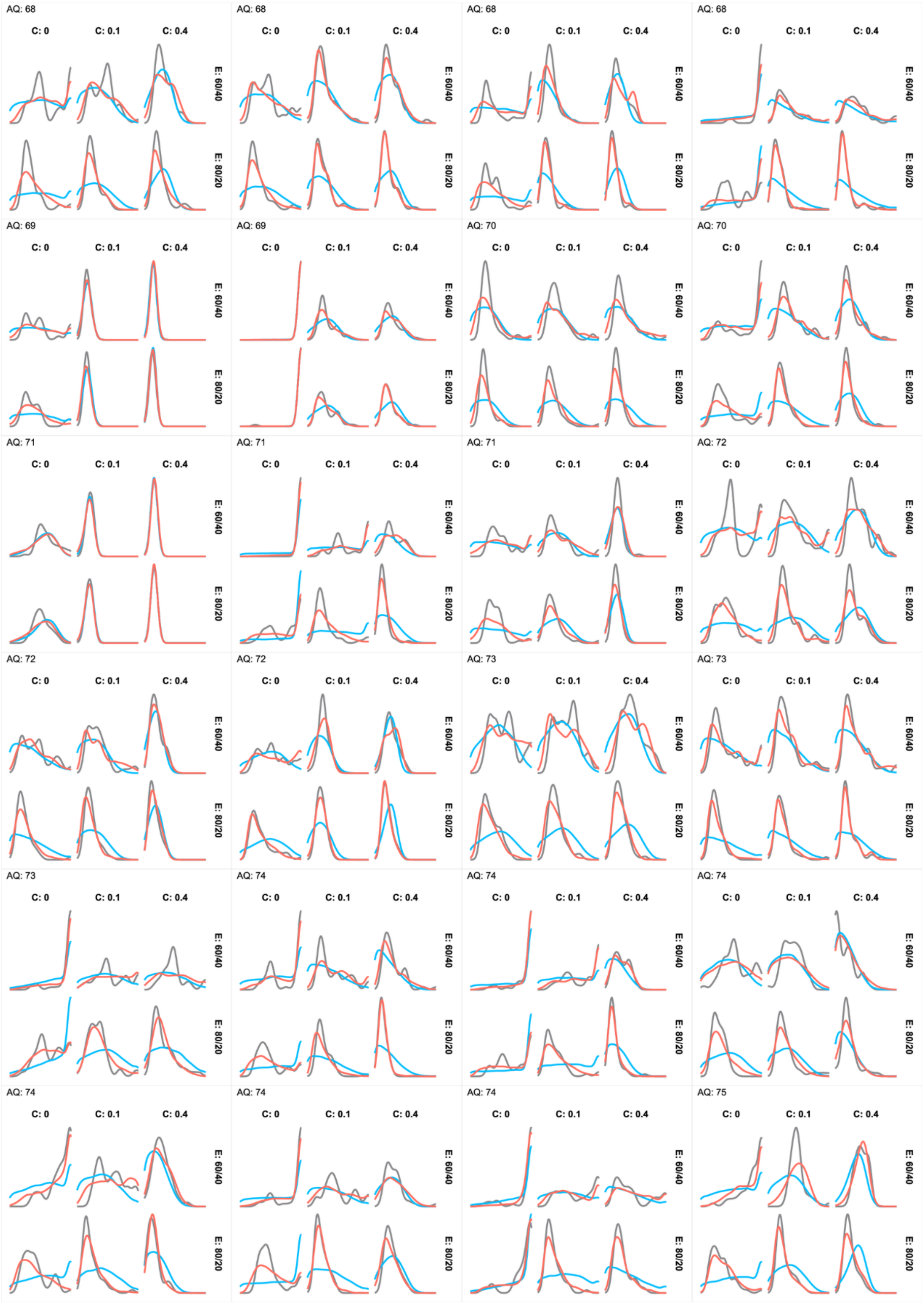
AQ: 68
C: 0
C: 0.1
C: 0.4
E: 60/40
E: 80/20
AQ: 68
C: 0
C: 0.1
C: 0.4
E: 60/40
E: 80/20
AQ: 68
C: 0
C: 0.1
C: 0.4
E: 60/40
E: 80/20
AQ: 68
C: 0
C: 0.1
C: 0.4
E: 60/40
E: 80/20
AQ: 69
C: 0
C: 0.1
C: 0.4
E: 60/40
E: 80/20
AQ: 69
C: 0
C: 0.1
C: 0.4
E: 60/40
E: 80/20
AQ: 70
C: 0
C: 0.1
C: 0.4
E: 60/40
E: 80/20
AQ: 70
C: 0
C: 0.1
C: 0.4
E: 60/40
E: 80/20
AQ: 71
C: 0
C: 0.1
C: 0.4
E: 60/40
E: 80/20
AQ: 71
C: 0
C: 0.1
C: 0.4
E: 60/40
E: 80/20
AQ: 71
C: 0
C: 0.1
C: 0.4
E: 60/40
E: 80/20
AQ: 72
C: 0
C: 0.1
C: 0.4
E: 60/40
E: 80/20
AQ: 72
C: 0
C: 0.1
C: 0.4
E: 60/40
E: 80/20
AQ: 72
C: 0
C: 0.1
C: 0.4
E: 60/40
E: 80/20
AQ: 73
C: 0
C: 0.1
C: 0.4
E: 60/40
E: 80/20
AQ: 73
C: 0
C: 0.1
C: 0.4
E: 60/40
E: 80/20
AQ: 73
C: 0
C: 0.1
C: 0.4
E: 60/40
E: 80/20
AQ: 74
C: 0
C: 0.1
C: 0.4
E: 60/40
E: 80/20
AQ: 74
C: 0
C: 0.1
C: 0.4
E: 60/40
E: 80/20
AQ: 74
C: 0
C: 0.1
C: 0.4
E: 60/40
E: 80/20
AQ: 74
C: 0
C: 0.1
C: 0.4
E: 60/40
E: 80/20
AQ: 74
C: 0
C: 0.1
C: 0.4
E: 60/40
E: 80/20
AQ: 74
C: 0
C: 0.1
C: 0.4
E: 60/40
E: 80/20
AQ: 75
C: 0
C: 0.1
C: 0.4
E: 60/40
E: 80/20

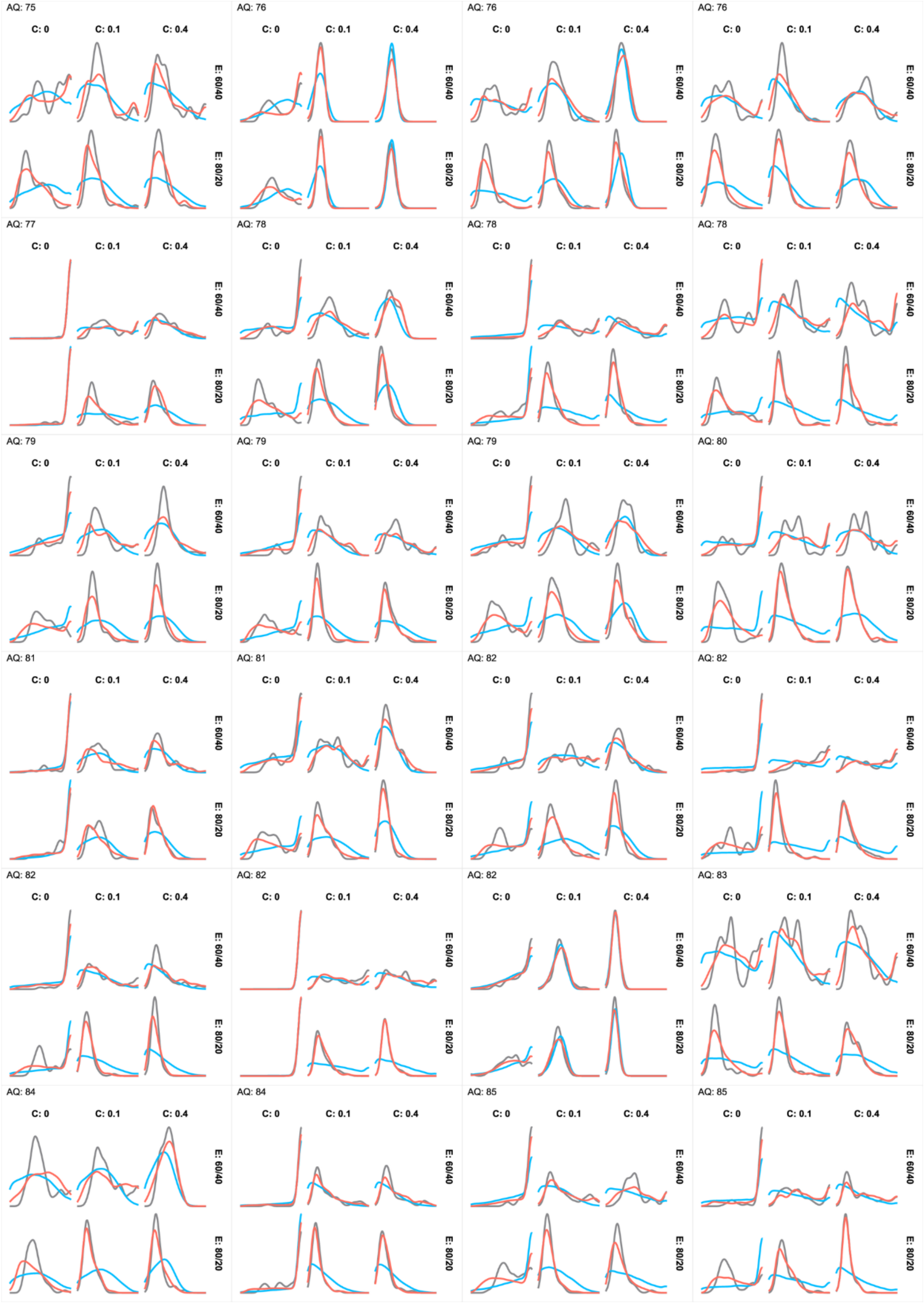
AQ: 75
C: 0
C: 0.1
C: 0.4
E: 60/40
E: 80/20
AQ: 76
C: 0
C: 0.1
C: 0.4
E: 60/40
E: 80/20
AQ: 76
C: 0
C: 0.1
C: 0.4
E: 60/40
E: 80/20
AQ: 76
C: 0
C: 0.1
C: 0.4
E: 60/40
E: 80/20
AQ: 77
C: 0
C: 0.1
C: 0.4
E: 60/40
E: 80/20
AQ: 78
C: 0
C: 0.1
C: 0.4
E: 60/40
E: 80/20
AQ: 78
C: 0
C: 0.1
C: 0.4
E: 60/40
E: 80/20
AQ: 78
C: 0
C: 0.1
C: 0.4
E: 60/40
E: 80/20
AQ: 79
C: 0
C: 0.1
C: 0.4
E: 60/40
E: 80/20
AQ: 79
C: 0
C: 0.1
C: 0.4
E: 60/40
E: 80/20
AQ: 79
C: 0
C: 0.1
C: 0.4
E: 60/40
E: 80/20
AQ: 80
C: 0
C: 0.1
C: 0.4
E: 60/40
E: 80/20
AQ: 81
C: 0
C: 0.1
C: 0.4
E: 60/40
E: 80/20
AQ: 81
C: 0
C: 0.1
C: 0.4
E: 60/40
E: 80/20
AQ: 82
C: 0
C: 0.1
C: 0.4
E: 60/40
E: 80/20
AQ: 82
C: 0
C: 0.1
C: 0.4
E: 60/40
E: 80/20
AQ: 82
C: 0
C: 0.1
C: 0.4
E: 60/40
E: 80/20
AQ: 82
C: 0
C: 0.1
C: 0.4
E: 60/40
E: 80/20
AQ: 82
C: 0
C: 0.1
C: 0.4
E: 60/40
E: 80/20
AQ: 83
C: 0
C: 0.1
C: 0.4
E: 60/40
E: 80/20
AQ: 84
C: 0
C: 0.1
C: 0.4
E: 60/40
E: 80/20
AQ: 84
C: 0
C: 0.1
C: 0.4
E: 60/40
E: 80/20
AQ: 85
C: 0
C: 0.1
C: 0.4
E: 60/40
E: 80/20
AQ: 85
C: 0
C: 0.1
C: 0.4
E: 60/40
E: 80/20

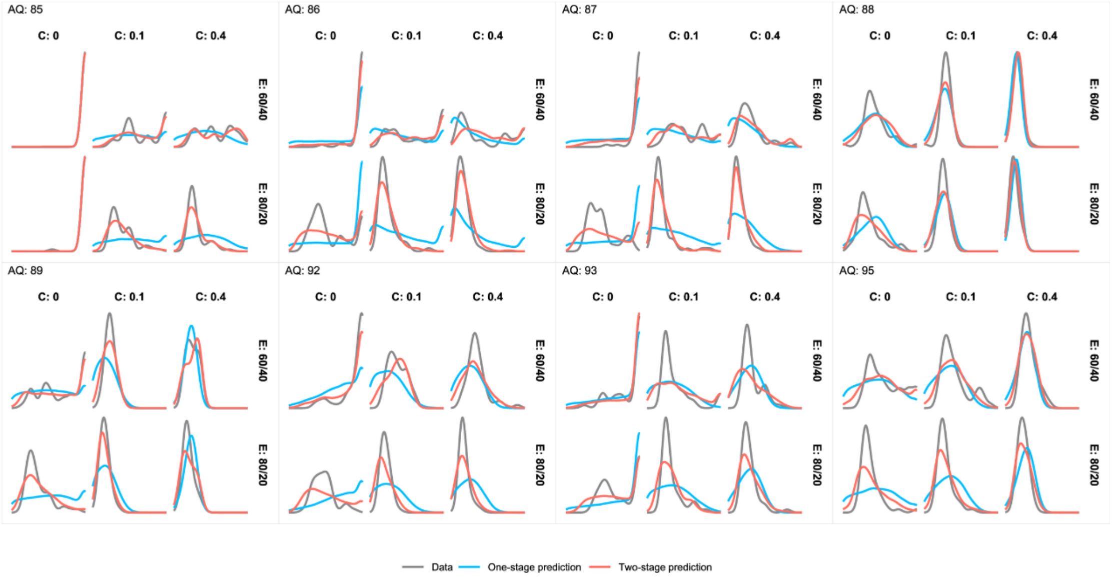

S6 Fig. Sample size distributions of individual participants in each cost and evidence condition: data vs. model predictions (extended to 5 pages).

Gray, blue, and red lines respectively denote data, the best one-stage model predictions, and the best two-stage model predictions. Each panel is for one participant, with each of its sub-panels for one cost and evidence condition. Panels are arranged by participants' AQ (marked at the top-left corner) ascendingly from left to right and from top to bottom. For most participants, the observed sample size distributions were better predicted by the best-fit one-stage model than by the best-fit one-stage model. C: Cost, E: Evidence.
