# Supplementary material for: Autistic traits influence the strategic diversity of information sampling: Insights from two-stage decision models
Source: PLoS Comput Biol. 2019 Dec 2;15(12):e1006964. doi: 10.1371/journal.pcbi.1006964 (PMC6907874; doi:10.1371/journal.pcbi.1006964)
Supplement: S7 Fig — There were little correlations between cost-evidence strategy index (AICccost→evidence−AICcevidence→cost) and participants’ age (a) or IQ score (b); cost-evidence strategy index did not differ between genders either (c). (PDF) [file pcbi.1006964.s008.pdf]

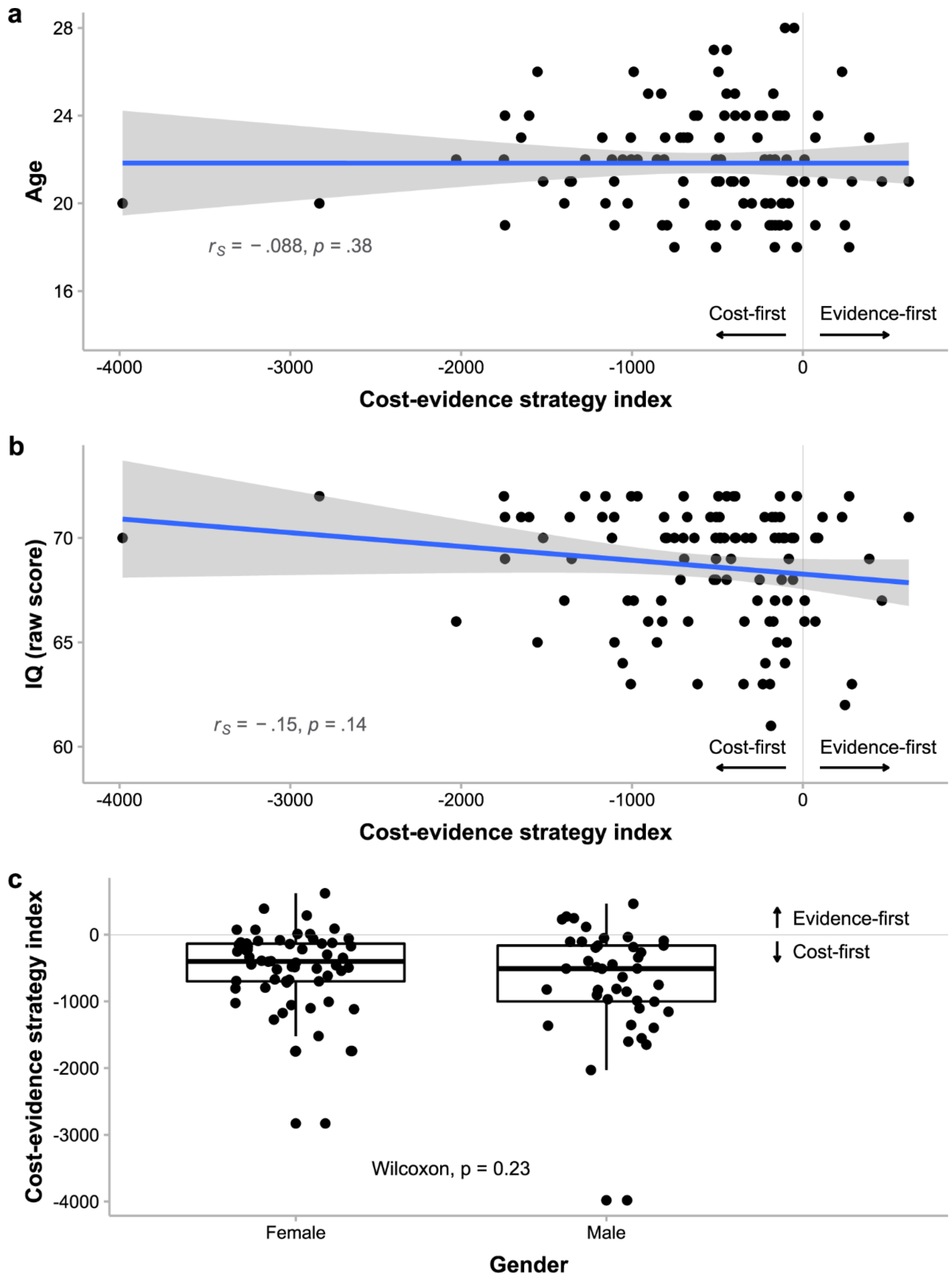

S7 Fig. The use of cost-first vs. evidence-first decision did not relate to age, IQ, or gender. There were little correlations between participants' cost-evidence strategy index ($AICc_{\text{cost}\rightarrow\text{evidence}} - AICc_{\text{evidence}\rightarrow\text{cost}}$) and their age (a) or IQ score (b); cost-evidence strategy index did not differ between genders either (c).
