# Supplementary material for: Autistic traits influence the strategic diversity of information sampling: Insights from two-stage decision models
Source: PLoS Comput Biol. 2019 Dec 2;15(12):e1006964. doi: 10.1371/journal.pcbi.1006964 (PMC6907874; doi:10.1371/journal.pcbi.1006964)
Supplement: S8 Fig — Correlations between cost-evidence strategy index (AICccost→evidence−AICcevidence→cost) and sampling bias (signed deviation from the optimal number of sampling, denoted ns−nopt¯) or sampling variation (standard deviation of actual number of sampling across trials, denoted SD(ns)) under different cost and evidence conditions were consistent with what we would expect if AQ affects these measures through cost-first vs. evidence-first preference in decision process. See Fig 6B for the corresponding plot for efficiency. Error bars represent 95% confidence intervals (FDR corrected). C:0 = zero-cost, C:0.1 = low-cost, C:0.4 = high-cost, E:0.6 = low-evidence, E:0.8 = high-evidence. (PDF) [file pcbi.1006964.s009.pdf]

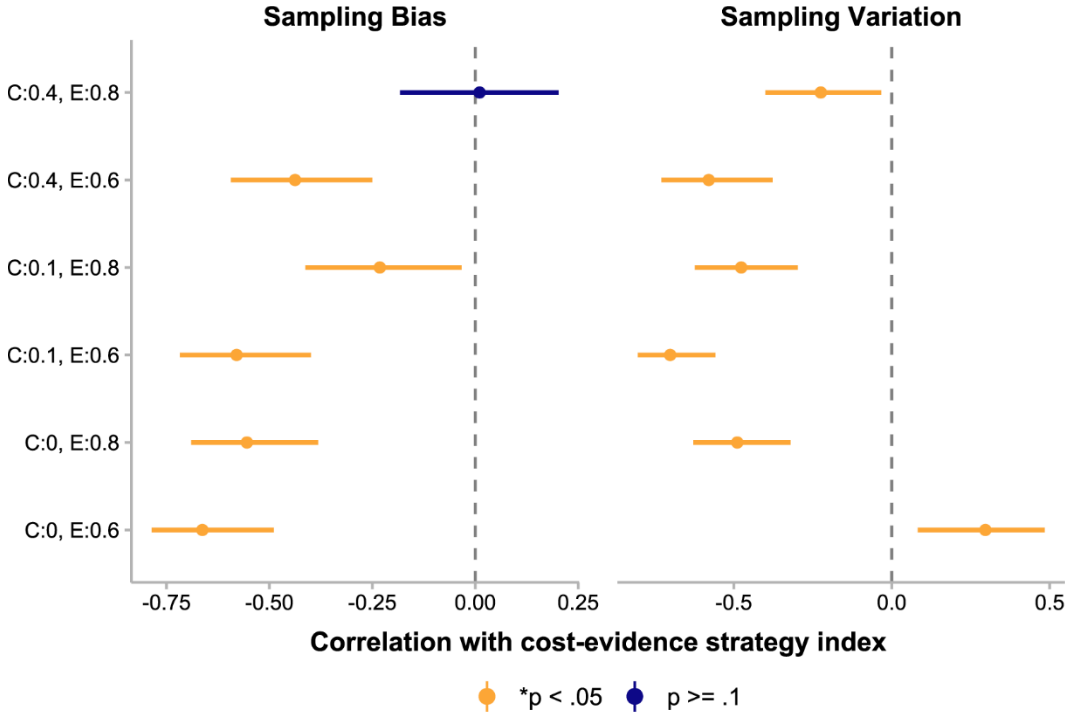

S8 Fig. How the use of cost-first vs. evidence-first decision processes related to sampling bias and sampling variation.

Correlations between cost-evidence strategy index ($AICc_{\text{cost}\rightarrow\text{evidence}} - AICc_{\text{evidence}\rightarrow\text{cost}}$) and sampling bias (signed deviation from the optimal number of sampling, denoted $\overline{n_s - n_{opt}}$) or sampling variation (standard deviation of actual number of sampling across trials, denoted $SD(n_s)$) under different cost and evidence conditions were consistent with what we would expect if AQ affects these measures through cost-first vs. evidence-first preference in decision process. See Fig 6b for the corresponding plot for efficiency. Error bars represent 95% confidence intervals (FDR corrected). C:0 = zero-cost, C:0.1 = low-cost, C:0.4 = high-cost, E:0.6 = low-evidence, E:0.8 = high-evidence.
