# Supplementary figures and images for: Autistic traits influence the strategic diversity of information sampling: Insights from two-stage decision models
Source: PLoS Comput Biol. 2019 Dec 2;15(12):e1006964. doi: 10.1371/journal.pcbi.1006964 (PMC6907874; doi:10.1371/journal.pcbi.1006964)

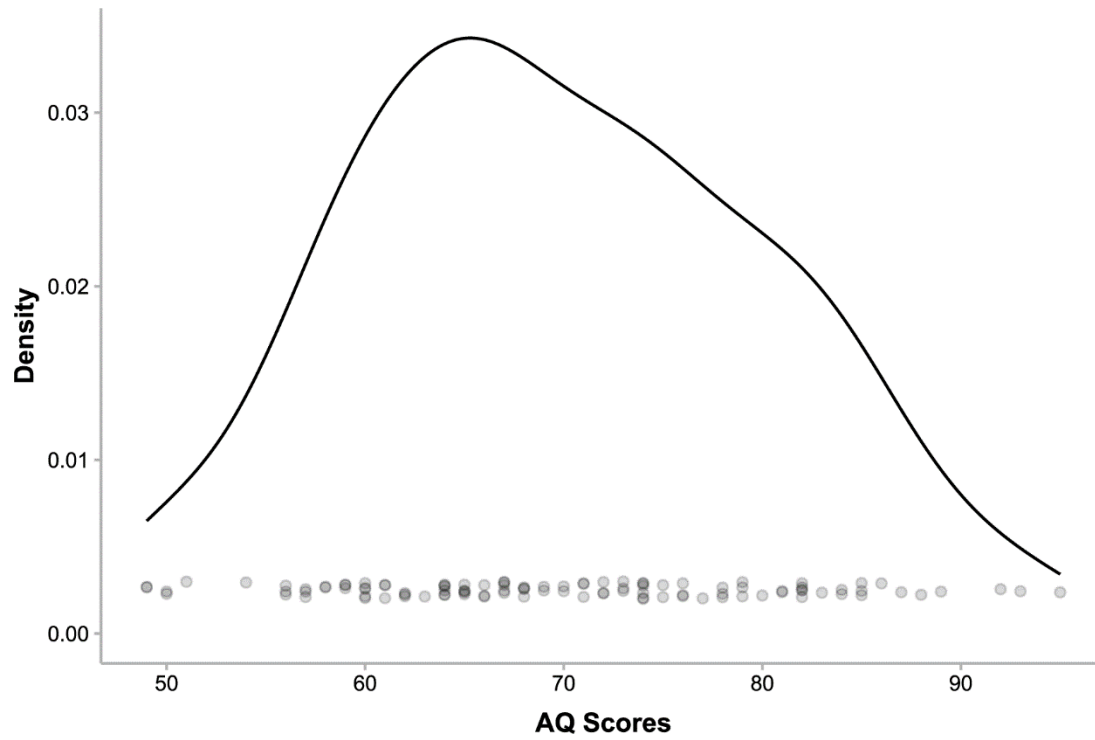

**S9 Fig. Distribution of AQ scores among the 104 participants.** Each circle denotes one participant.

Supplement: S9 Fig — Each circle denotes one participant. (PDF) [file pcbi.1006964.s010.pdf]
