# Supplementary material for: Autistic traits influence the strategic diversity of information sampling: Insights from two-stage decision models
Source: PLoS Comput Biol. 2019 Dec 2;15(12):e1006964. doi: 10.1371/journal.pcbi.1006964 (PMC6907874; doi:10.1371/journal.pcbi.1006964)
Supplement: S10 Fig — Following Jones et al. [81], we identified three observations (red dots) as “likely noncompliant” in the number of bead samples for each condition based on nonparametric boxplot statistics, that is, those whose values were lower than the 1st quartile or higher than the 3rd quartile of all the observations in the condition by more than 1.5 times of the interquartile range. These observations (not participants per se) were excluded from linear mixed model analyses 1–3 (LMM 1–3, see Methods). (PDF) [file pcbi.1006964.s011.pdf]

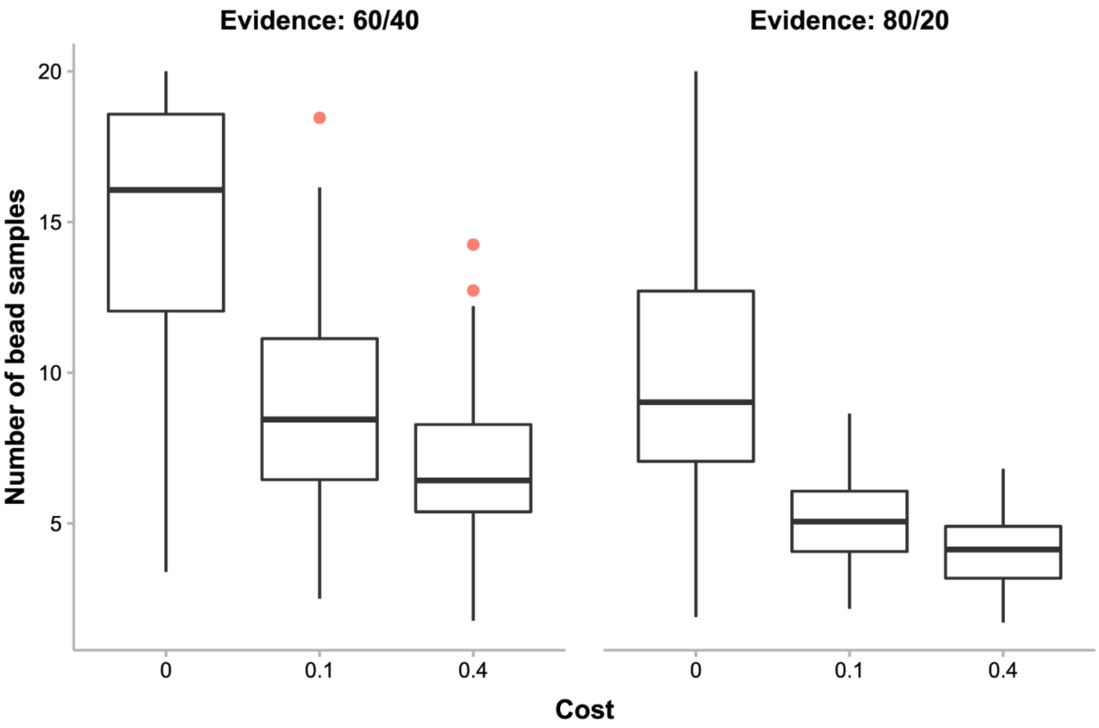

S10 Fig. Noncompliant observations.

Following Jones et al. [81], we identified three observations (red dots) as “likely noncompliant” in the number of bead samples for each condition based on nonparametric boxplot statistics, that is, those whose values were lower than the 1st quartile or higher than the 3rd quartile of all the observations in the condition by more than 1.5 times of the interquartile range. These observations (not participants per se) were excluded from linear mixed model analyses 1-3 (LMM 1-3, see Methods).
